# Enhancing Flame-Retardant Properties of Polyurethane Aerogels Doped with Silica-Based Particles

**DOI:** 10.3390/gels10070465

**Published:** 2024-07-16

**Authors:** Esther Pinilla-Peñalver, Óscar del Fresno, Darío Cantero, Adriana Moreira, Filipa Gomes, Francisca Miranda, Marcelo Oliveira, Mariana Ornelas, Luz Sánchez-Silva, Amaya Romero

**Affiliations:** 1 Department of Chemical Engineering, University of Castilla-La Mancha, Avda. Camilo José Cela 12, 13071 Ciudad Real, Spain; 2 CeNTI—Centre for Nanotechnology and Smart Materials, Rua Fernando Mesquita 2785, 4760-034 V. N. Famalicão, Portugal; amoreira@centi.pt (A.M.);

**Keywords:** environmentally friendly fillers, flame-retardant properties, polymer materials, polyurethane aerogels, reinforcement, silica particles

## Abstract

**Highlights:**

Aerogels doped with SiO_2_ particles derived from rice husks are obtained by a freeze-drying method.

SiO_2_ particles provide a reduced-flammability advantage over undoped PUR aerogels.

Increasing SiO_2_ content significantly enhances flame-retardancy in PUR aerogels.

**Abstract:**

In this work, polyurethane (PUR) aerogels doped with different SiO_2_ particles, derived from a renewable source, were successfully synthesized, and the effects of SiO_2_ content on the properties of PUR aerogels were investigated. Specifically, three types of SiO_2_-based particles obtained from rice husk through different procedures were evaluated to enhance the thermal stability of the composites with special attention given to flame-retardant properties. With the optimal SiO_2_ particles, obtained through acid digestion, the influence of their content between 0.5 and 3 wt.% on the physicochemical characteristics of the synthesized aerogels was thoroughly examined. The results showed that increasing the doping agent content improved the lightness, thermal stability, and flame-retardant properties of the resulting PUR aerogels, with the best performance observed at a 2 wt.% doping level. The doped aerogel samples with non-modified SiO_2_ particles significantly enhanced the fire safety performance of the material, exhibiting up to an eightfold increase in flame retardancy. However, modification of the SiO_2_ particles with phytic acid did not slow down the combustion velocity when filling the aerogels. This research highlights the promising potential of doped PUR/SiO_2_ aerogels in advancing materials science and engineering applications for withstanding high temperatures and improving fire safety.

## 1. Introduction

Making buildings more efficient in terms of fire resistance in construction materials is essential to minimize fire spread and extend evacuation time. Incorporating fire-resistant materials, such as insulation panels and coatings, is key in slowing down flame propagation and reducing the emission of harmful toxic smoke [1].

Conventional insulation materials, such as foams (colloidal systems like sols, gels, and aerogels), are widely used for insulation and have advantages such as being widely available, affordable, and easy to install. However, they also have disadvantages such as low fire resistance (requiring the incorporation of flame-retardant additives), potential toxic emissions, and limited energy efficiency. In this sense, it is important to note that combustible insulation materials are responsible for most fatal fires. The widespread use of these types of commercial materials has generated an urgent need to develop safer and more efficient alternatives, such as new fire-resistant insulation materials for buildings [2]. These materials are essential for preventing the spread of fire and protecting the lives of building occupants. Ongoing technological advancements and research into new materials are leading to the development of safer and more efficient options in terms of fire resistance, providing enhanced protection in case of emergencies. Depending on their nature, flame-retardant systems can act either physically (by cooling, forming a protective layer, or diluting fuel) or chemically (through reaction in the condensed or gas phase). They can interfere with the various processes involved in polymer combustion (heating, pyrolysis, ignition, and propagation of thermal degradation) [3,4].

Aerogels, highly porous materials with low density (sometimes likened to solid foams due to their structural similarities), are renowned for their exceptional thermal insulation properties [5]. Their historical and commercial significance spans nearly a century, during which they have undergone complex development but demonstrated remarkable potential across diverse applications [6,7]. Most aerogels are synthesized using the sol–gel method, a green synthesis technique that is applied under mild conditions and with eco-friendly solvents such as water and alcohols [8,9,10]. Recent focus has been on organic aerogels, particularly those primarily composed of polyurethane polymer chains (PUR aerogels), aimed at producing materials with exceptionally low conductivity and density [11,12,13]. Key attributes of organic aerogels include high insulation capacity, stemming from their low thermal conductivity effectively reducing heat transfer. Additionally, they boast low density and high porosity, affording a substantial surface area that enhances insulation performance. Furthermore, aerogels exhibit flexibility, enabling them to conform to various shapes and surfaces for convenient installation. However, the high flammability of most organic polymeric materials presents a significant challenge, resulting in the generation of substantial smoke, toxic gases, heat, and melting drips during combustion. Consequently, these factors contribute to considerable damage to human lives and properties annually, underscoring safety concerns across various fields of application [14].

To improve the fire-retardant behavior of PUR aerogels (and other hybrids synthesized via sol–gel in environmentally friendly conditions) and to reduce the release of plentiful heat and toxic smoke, different flame-retardant fillers can be incorporated into the aerogel matrix. These flame-retardant dopants should not negatively affect the synthesis process. Some additives that improve flame resistance and suppress smoke include carbon nanotubes [15], clays [16], lignin [17], phosphorus [18], and silica (SiO_2_) particles [19]. Among them, SiO_2_ particles stand out due to their small size and high surface area, which allow them to interact with the PUR matrix and enhance its structure and properties [20].

During the synthesis of SiO_2_-doped PUR aerogels, the concentration and dispersion of SiO_2_ particles, as well as the manufacturing process, can significantly influence the extent of property enhancement [21,22]. Generally, an increase in SiO_2_ content leads to enhanced mechanical strength, thermal stability, and fire resistance of the aerogel [19,23,24]. However, an excess of SiO_2_ particles can negatively affect the porosity and density of the aerogel, which can reduce its thermal insulation performance. Therefore, it is important to establish an appropriate balance in the content of SiO_2_ particles to achieve the best final properties of PUR aerogels [21,25].

The objective of this study was to investigate the influence of three types of SiO_2_ particles as doping agents in PUR aerogels, with a particular focus on flame-retardant properties. A novel aspect of this research is the use of SiO_2_ additives derived from natural rice husk (RH) as dopants, which are incorporated into the polymer matrix. This innovative approach not only reduces the environmental footprint by using a low-cost and widely available crop residue, but also enhances the sustainability of SiO_2_ production, contributing to the circular economy. The study details the effect of particle content on the physicochemical properties and performance of the synthesized aerogels.

## 2. Results and Discussion

### 2.1. Impact of Silica Particles’ Nature as Dopant of PUR Aerogels

The incorporation of SiO_2_ particles of a different nature depending on the extraction procedure (AD and SG at 2 wt.%) into the polymeric structure of PUR aerogels was evaluated in detail. Table 1 summarizes the SiO_2_-doped aerogels’ characterization results obtained in terms of density, thermal conductivity, Young’s modulus, and glass transition temperature (*T_g_*). The “PUR” sample corresponds to the aerogel sample that is free of SiO_2_ particles, commonly known as a blank sample.

As can be seen, the densities of the doped samples increase such that PUR_AD_2_ < PUR_SG_2_, while the low thermal conductivity values obtained for the doped PUR aerogels remain practically constant regardless of the type of particles incorporated. On the other hand, the incorporation of SiO_2__AD_2_ particles lightly increases the surface area and total pore volume of the resulting aerogel compared with the undoped one, contrary to what was observed when incorporating the SiO_2__SG_2_ particles, which resulted in aerogels with a very low surface area. As a result, higher density in the doped samples and lower surface area correspond to increased sample hardness, as indicated by Young’s modulus. The *T_g_* results for all samples at around −50 °C are consistent with the typical PUR structure [26].

**Figure 1:**
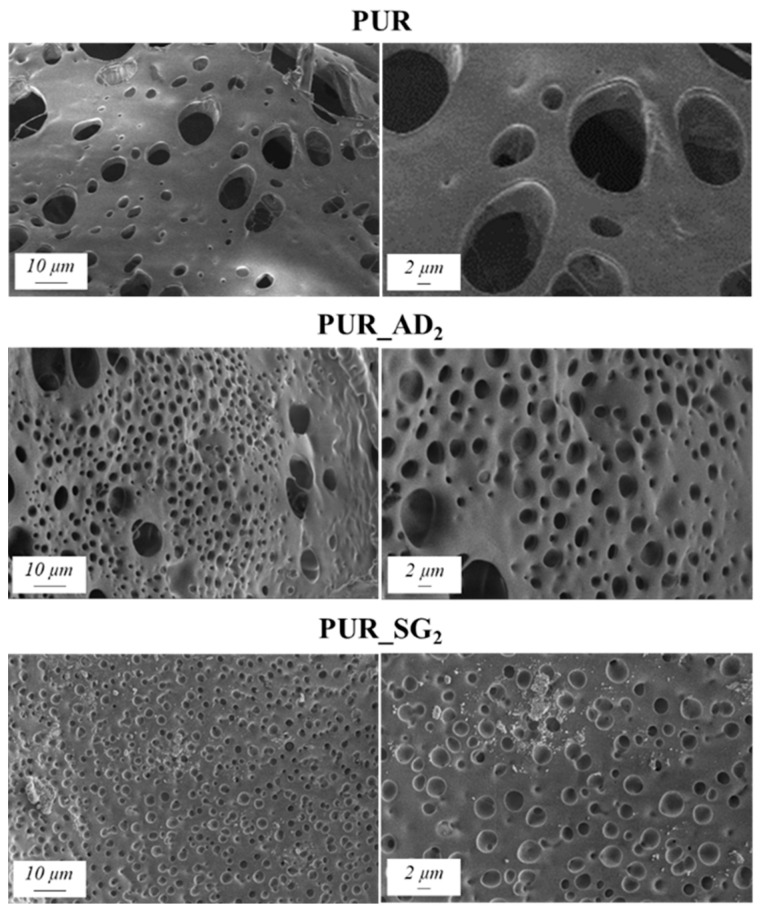
SEM micrographs corresponding to the synthesized SiO_2_-doped PUR aerogels.

**Figure 2:**
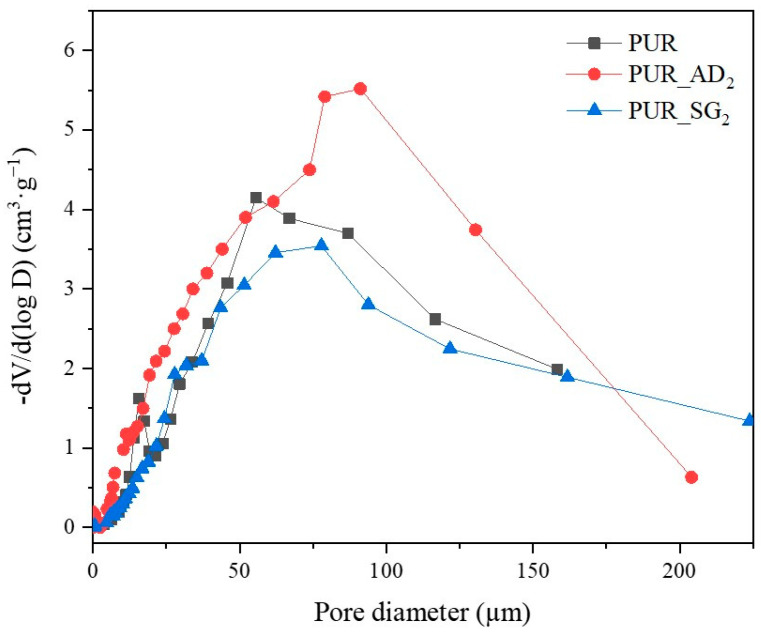
Pore size distribution of the synthesized SiO_2_-doped PUR aerogels.

The SEM results depicted in Figure 1 confirm the earlier findings. The porous structure of the PUR_SG_2_ aerogels is observed to be closing, resulting in a more compact appearance consistent with the surface area, pore volume, and density values already mentioned. This fact is further supported by the pore size distribution (PSD) obtained by a Hg porosimeter, which indicated pore sizes ranging from 3 to 300 µm for both samples, as illustrated in Figure 2. Moreover, as an example, the elemental mapping and EDS analysis of the PUR_AD_2_ sample are presented in Figure S1, showcasing the well-dispersed SiO_2_ particles throughout the polymer.

**Figure 3:**
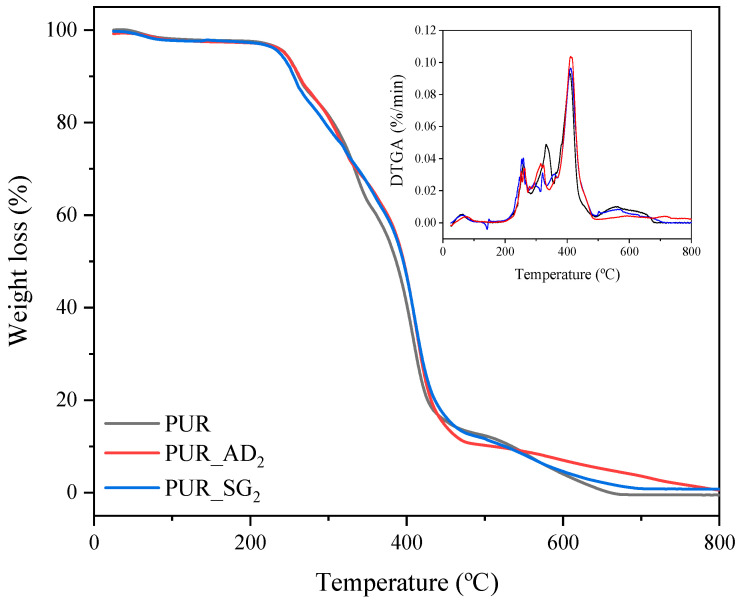
TGA and DTGA curves of the different SiO_2_-doped PUR aerogels.

The influence of the nature of the SiO_2_ particles incorporated into the structure of PUR aerogels on their thermal degradation is illustrated in Figure 3 (TGA curves corresponding to the synthesized SiO_2_-doped aerogel samples). As can be observed, similar profiles are found for the different aerogels, including the undoped one. PUR aerogel samples exhibit thermal stability of up to 230 °C, wherein they undergo complete loss of any water or residual solvents. Subsequently, between 230 and 440 °C, the most significant mass loss (>90 wt.%) occurs due to the decomposition of a PUR network. Finally, from 440 to 700 °C, the decomposition of ester groups takes place, leaving approximately 1 wt.% remaining [27,28].

**Figure 4:**
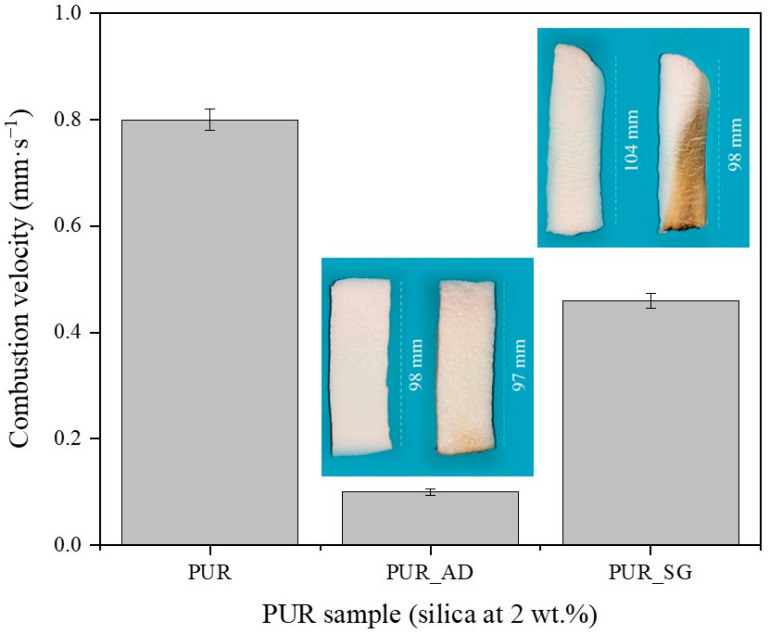
Combustion velocity of the synthesized SiO_2_-doped PUR aerogels (PUR_AD_2_ and PUR_SG_2_) and the corresponding pictures of PUR aerogels before and after the burning process.

To explore the use of synthesized SiO_2_-doped aerogels as fireproof materials in the construction sector, their flame-retardant capability was studied. The combustion velocity was calculated after the total time, ignition, and propagation (13 s) (Figure 4). As can be observed, both doped aerogels with AD and SG at 2 wt.% reduced the combustion velocity compared with the undoped aerogel. Interestingly, the presence of AD_2_ particles in the polymeric matrix significantly mitigated the combustion velocity of the aerogel, and it does so up to more than six times compared with PUR_SG_2_, resulting in a velocity as low as 0.07 ± 0.01 mm·s^−1^. Therefore, these results strongly suggest that SiO_2_ particles obtained through AD of RH and subsequent calcination (PUR_AD_2_) demonstrates superior performance of flame retardancy when acting as a doping agent for PUR compared with the sample obtained through alkaline extraction of the RH ash, followed by sol–gel precipitation (PUR_SG_2_). Considering the physicochemical characterization of the particles (Table 4), the results could be attributed to the fact that the alkaline extraction and sol–gel precipitation process generates SiO_2_ particles with the presence of metallic impurities, particularly alkaline oxides [29], which could be related to the formation of more compact and, therefore, more dense aerogels, as demonstrated previously. Nevertheless, it is noteworthy that acid leaching effectively removed most inorganic impurities, resulting in highly pure SiO_2_ (see FTIR results; Figure S2). In any case, the obtained flame-retardant results are in perfect agreement with the thermal stability observed from the thermogravimetric analyses conducted on each of the SiO_2_ particles (Figure S3). The combustion of polymeric materials initiates upon the application of heat, leading to thermal degradation. The resultant degradation byproducts become superheated and congregate to create bubbles. One strategy for flame retardancy involves reducing the rate of bubbling. In this sense, SiO_2_ particles reduce the flammability of polymer materials by suppressing the intense bubbling phenomenon that occurs during the degradation process in combustion [30,31].

Based on the achieved results, the SiO_2__AD_2_ particle type was selected as a dopant for PUR aerogels with the aim of enhancing the flame-retardant properties of the resulting materials.

### 2.2. Effect of the Content of Acid-Extracted SiO_2_ Particles in PUR Aerogels

After selecting AD particles as the best SiO_2_ doping agent, the impact of their concentration in the aerogels was evaluated within the range of 0.5 to 3 wt.%. The findings related to density, thermal conductivity, and Young’s modulus are outlined in Table 2.

**Figure 5:**
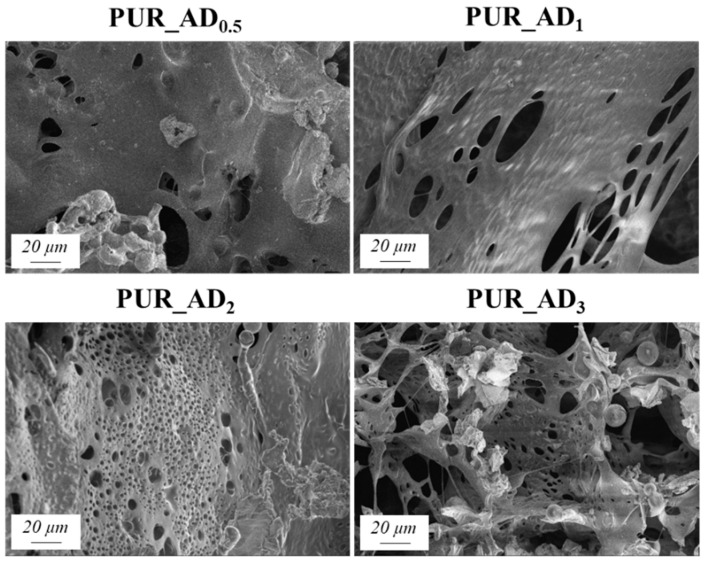
SEM micrographs corresponding to the different PUR_AD aerogels.

**Figure 6:**
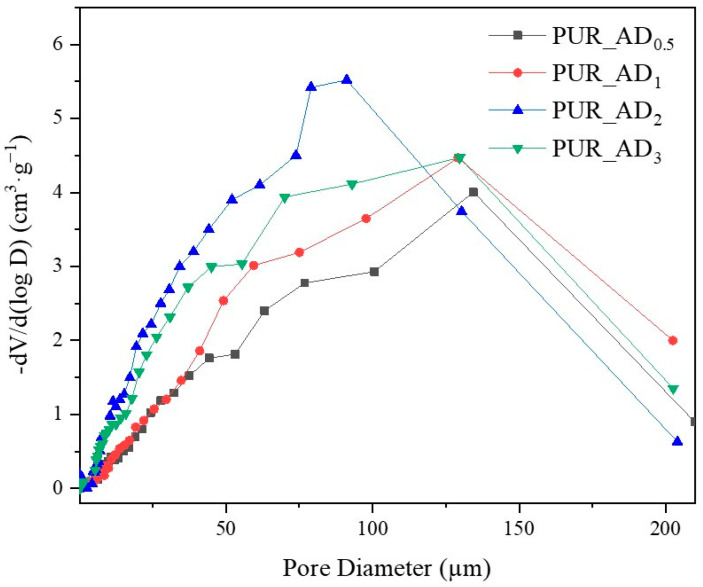
Pore size distribution corresponding to the different PUR_AD aerogels.

The correlation between SiO_2_ content and density values is evident, supported by observations from SEM micrographs (Figure 5). These images reveal increased porosity at a higher dopant content, leading to a more porous structure in samples with over 0.5% SiO_2_ content. This contrasts with the denser nature of the 0.5 wt.% doped sample, as confirmed by density results. As anticipated, lighter samples (PUR_AD_2_ and PUR_AD_3_) indicate a higher surface area and greater total intruded pore volume. However, it is remarkable that the PUR_AD_2_ sample contains a higher number of small-sized pores compared with the PUR_AD_3_ aerogel, which has an open surface and lacks large-sized pores. This fact can be corroborated in Figure 6, where the pore size distributions (PSDs) of PUR aerogels doped with SiO_2__AD particles at different percentages are represented. The PSD shows how the peak of the Gaussian representation shifts to lower values as the metallic content increases, until it reaches approximately 2%.

Moreover, although the thermal conductivity values seem to remain relatively unchanged across the doped samples, all of them exhibit enhanced insulating properties compared with the undoped sample. Furthermore, an increase in SiO_2_ content corresponds to a rise in Young’s modulus, which was determined from the linear region of the stress–strain curves, indicating an improvement in the mechanical properties of the samples [32].

**Figure 7:**
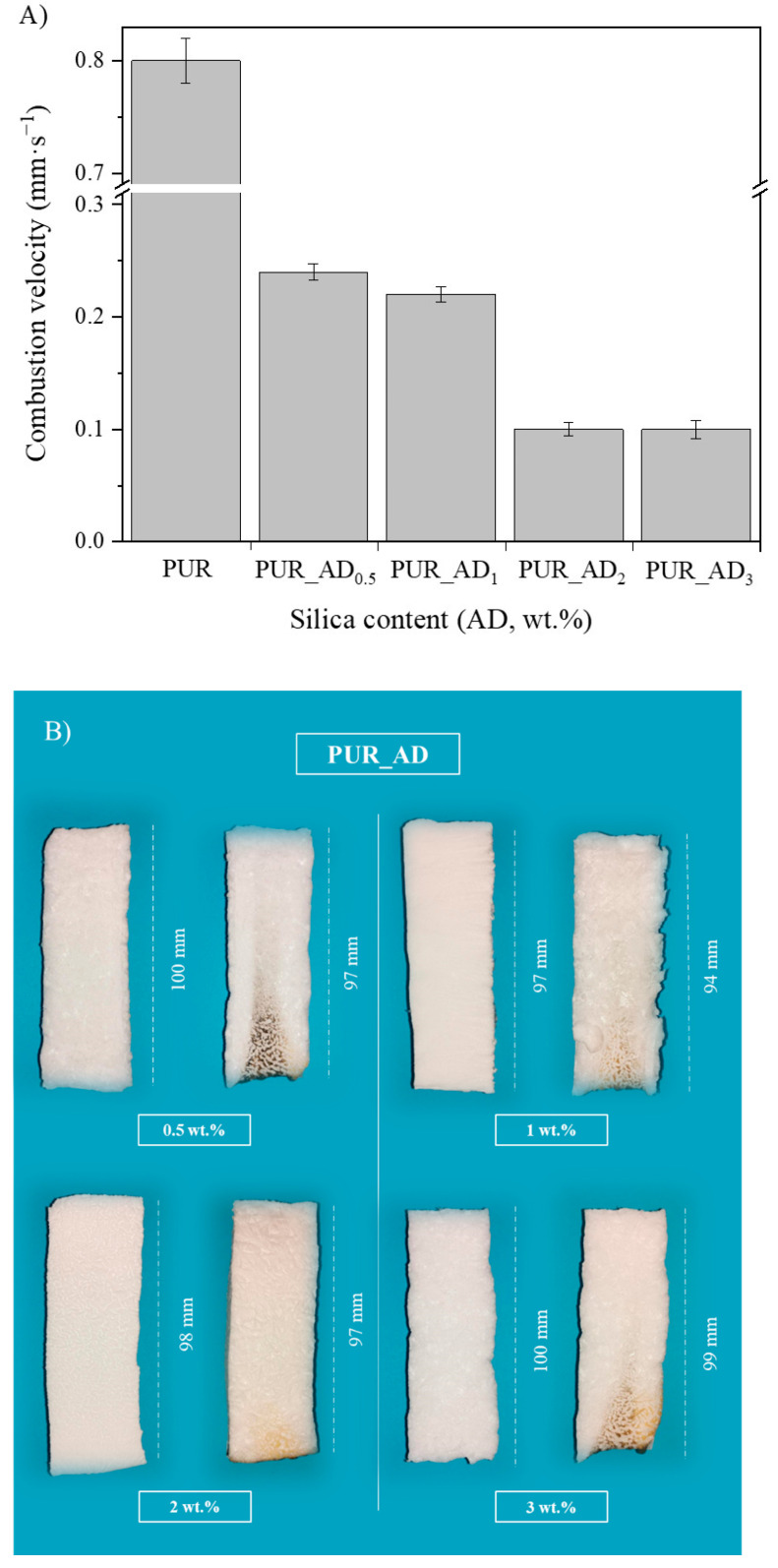
(**A**) Combustion velocity corresponding to the aerogel synthesized using different wt.% of AD_SiO_2_ particles; (**B**) pictures of polyurethane aerogels before (left) and after (right) the burning process.

The study aimed to evaluate the influence of SiO_2_ content on fire safety enhancement. Specifically, the impact of SiO_2_ content on PUR aerogels was examined in relation to combustion velocity (Figure 7A). Experimental images of the residual char post-sample combustion are presented in Figure 7B. The results indicate an inverse relationship between SiO_2_ content and combustion rate within the studied range. However, a SiO_2_ content exceeding 2 wt.% does not result in a reduction in combustion velocity. According to the results, it seems that the improvement in the flame-retardant properties of the synthesized aerogels is closely connected to the porosity of the samples. According to the literature for other fillers [33], aerogels doped with SiO_2_ fillers exhibited smaller sizes and more uniform pores (see Figure 1 and Figure 5, PUR undoped vs. PUR_AD_2_). This was attributed to the addition of fillers increasing the viscosity of the precursor solution, acting as nucleation agents. This contributed to an ordered structure, resulting in enhanced mechanical and thermal properties, thereby making flame propagation more challenging. Therefore, the comparable combustion speeds of the PUR_AD_2_ and PUR_AD_3_ samples are attributed to their similar surface area and pore volume, as previously mentioned, despite their differing morphologies. Therefore, a SiO_2_ content of 2 wt.% of SiO_2__AD is deemed the optimal particle concentration for incorporation into the polymer structure to minimize the flammability of aerogel samples. Following common practice in the literature for this type of materials [34,35], different images were recorded with a thermal camera where the sample emits an amount of infrared radiation as a function of its temperature (Figure S4). The images reveal the notable difference in combustion when the samples contain a SiO_2_-based additive (2 wt.%) extracted by AD from RH, showing the char residues generated in each case. PUR undergoes thermal degradation through a well-known mechanism [36,37] that involves its dissociation into primary amines, olefins, and carbon dioxide. This process typically occurs when PUR is exposed to elevated temperatures, leading to the breakdown of its polymeric structure (Figure S5). With the addition of a SiO_2_-based additive, the samples tend to form an intumescent char layer after combustion. The high quality of a SiO_2_ layer form would act as a good physical barrier to resist the transfer of heat and combustible gases, resulting in the enhancement of flame-retardant performance. However, as other authors have observed, SiO_2_ excess could suppress the intumescent process of the carbonization layer, resulting in a decrease in the synergistic effect on the flame retardancy and smoke suppression properties of the coatings [38].

The combustion study suggests that enhancing the fire resistance of samples can be feasibly achieved by incorporating additives derived from natural and cost-effective sources, such as SiO_2_ particles obtained from RH, as opposed to their respective counterparts.

### 2.3. Effect of the Modification of SiO_2_ Particles with a Flame-Retardant Agent

It is widely described in the literature that PA is a bio-based and environmentally friendly compound that is used as a flame-retardant agent in different matrices [39,40]. By using QAS as a crosslinker, an effective anchoring of PA to SiO_2__AD particles was achieved (as demonstrated by FTIR analysis; see Figure S2).

**Figure 8:**
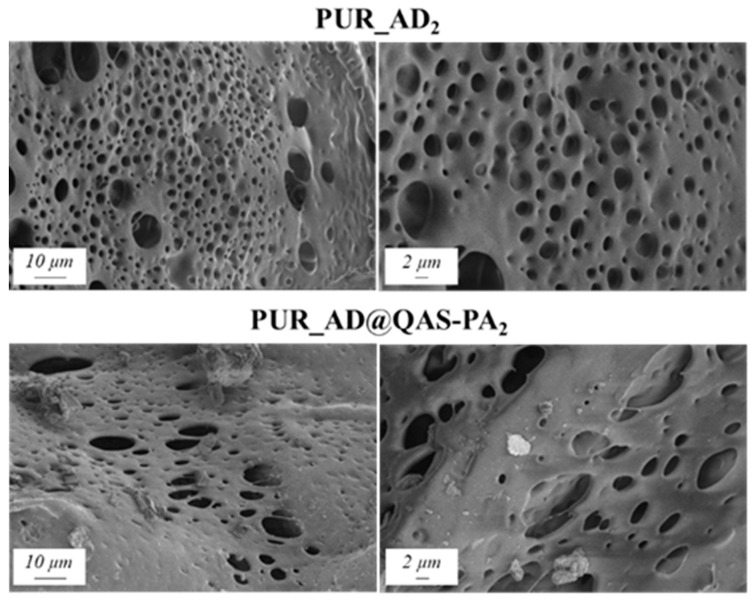
SEM micrographs corresponding to PUR aerogels doped with SiO_2_ particles derived from acidic extraction.

In agreement with the findings reported in the previous subsection, the selected content of AD@QAS-PA for the preparation of PUR samples was 2 wt.%. Physicochemical properties (Table 3) and the SEM results (Figure 8) show that the incorporation of SiO_2__AD@QAS-PA_2_ particles resulted in aerogels with a very low surface area and high density. As a result, this increased the aerogel hardness, as indicated by Young’s modulus. The porous structure of the PUR_AD@QAS-PA_2_ aerogels is observed to be closing, resulting in a more compact appearance consistent with the surface area, pore volume, and density values already mentioned.

**Figure 9:**
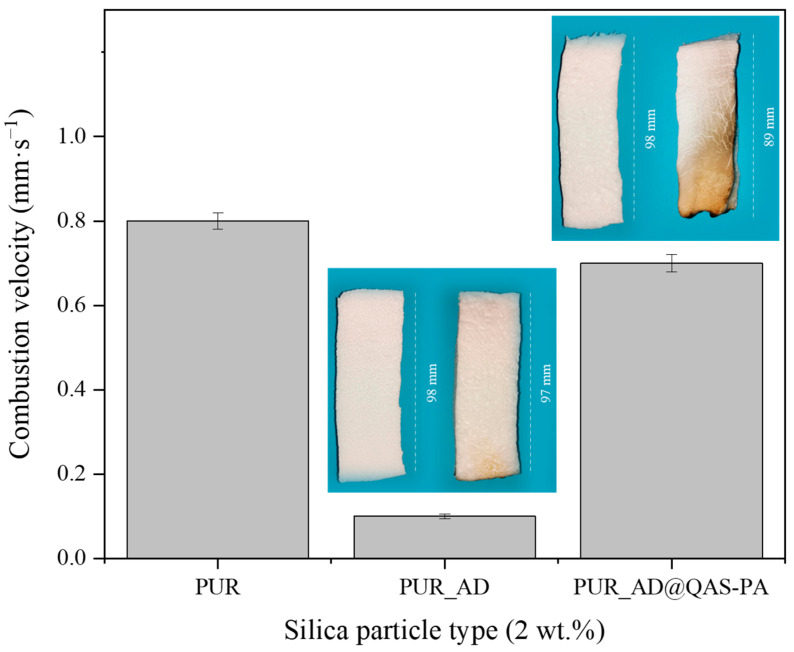
Combustion velocity of the synthesized SiO_2_-doped PUR aerogels (PUR_AD_2_ and PUR_AD@QAS-PA_2_) and the corresponding pictures of PUR aerogels before and after the burning process.

Figure 9 shows the calculated combustion velocity of the doped aerogels with AD and AD@QAS-PA particles. Unexpectedly, the incorporation of AD@QAS-PA particles into the polymeric matrix practically does not significantly slow down the combustion velocity of the sample, showing slightly lower values but very close to those of the undoped aerogel (PUR). This observation contradicts the expected improvement of the AD particles modification in the flame-retardant behavior according to other authors [39,40]. This discrepancy likely arises from the density of the modified particles. Specifically, since the modified particles are denser than their non-modified counterparts (as indicated in Table 1 and Table 3) due to the long alkyl silane chain of QAS, a smaller quantity of AD@QAS-PA particles fills the matrix when compared with the same mass of SiO_2__AD. Consequently, this minimizes the potential flame-retardant effect.

To gain further insights into the flame-retardant behavior of doped aerogels, additional studies—particularly those considering an addition of SiO_2_ dopants in a weight/volume ratio instead of a weight/weight ratio—should be conducted to explore the effective impact of using the modified biosilica in the polymeric matrix. In any case, this study suggests that enhancing the fire resistance of samples can be feasibly achieved by incorporating additives derived from natural and cost-effective sources, such as SiO_2_ particles obtained from RH, as opposed to their respective counterparts.

## 3. Conclusions

A novel method is proposed to produce polyurethane/silica (PUR/SiO_2_) aerogel composites with evenly dispersed SiO_2_ particles. This innovation utilizes SiO_2_ particles obtained from sustainable sources like rice husk through different procedures, introducing them as doping agents into the polymer structure of PUR aerogels. The physicochemical properties of both undoped and doped aerogels with different types of SiO_2_ particles (AD and SG) were analyzed, resulting in mechanically strengthened materials across the board. Additionally, the flammability of aerogels was investigated, showing a notable reduction in the burning rate, especially for aerogels doped with SiO_2_ particles extracted from acid digestion (AD). The influence of particle content on the combustion rate was confirmed, with samples containing over 2 wt.% displaying similar thermal conductivity, density, and porosity results without further enhancement. However, the functionalization of the AD particles with PA did not delay flame propagation. In summary, the doped aerogel samples significantly enhance the fire safety performance of the material, which is crucial in various sectors such as construction and automotive, with up to an eightfold increase in fire resistance when only doped with 2 wt.% of AD particles. Future work could focus on optimizing the doping process and exploring other sustainable sources for SiO_2_ particles, as well as investigating additional functionalization techniques to further improve the flame-retardant properties and overall performance of the aerogels.

## 4. Materials and Methods

### 4.1. Reagents and Materials

For hydrogel preparation: 4,4′-methylenebis(cyclohexyl isocyanate) [HMDI, 90% mixture of isomers, Sigma-Aldrich, St. Louis, MO, USA]; polyethylene glycol [PEG, number-average molecular weight (Mn) = 2000 g·mol^−1^, Sigma-Aldrich]; 2,2-bis(hydroxymethyl)propionic acid [DMPA, 98%; Aldrich Chemicals]; 1-methyl-2-pyrrolidinone anhydrous [NMP, 99.5%, Sigma-Aldrich)]; dibutyltin dilaurate [DBTDL, 95%, Aldrich Chemicals, Milwaukee, WI, USA]; triethylamine [TEA, ≥99%, Sigma-Aldrich]; ethylenediamine [EDA, ≥99%, Sigma-Aldrich]; and ethyl acetate anhydrous [EtOAc, 99.8%, PanReac AppliChem, Barcelona, Spain]. Water was purified by distillation, followed by deionization using ion-exchange resins (resistivity 18.2 MΩ·cm). PEG and DMPA were dried overnight at 60 °C under vacuum before use to remove residual water, while other chemicals were used as acquired.

For the SiO_2_ particles extraction from rice husk: rice husk [RH, Arrozeiras Mundiarroz, S.A., Coruche, Portugal], nitric acid [69%, PanReac AppliChem, Chicago, IL, USA], sodium hydroxide [Pronalab, Lisbon, Portugal], and hydrochloric acid [37% *v*/*v*, Fischer Scientific, Madrid, Spain] were used.

For the modification of SiO_2_ particles: dimethyloctadecyl(3-(trimethylsilyl)propyl ammonium chloride solution [QAS, 42 wt.%, Sigma-Aldrich], phytic acid [PA, 50 wt.%, Fischer Scientific], and absolute ethanol [EtOH, Aga].

### 4.2. Synthesis Procedures

#### 4.2.1. Extraction of SiO_2_ Particles

The SiO_2_ particles were obtained by acidic digestion of RH or by alkaline extraction of the husk ash, following procedures widely described in the literature [41]. Both the acidic and alkaline processes yielded approximately 10% of SiO_2_. The extraction was performed as follows:

- Acidic digestion: HNO_3_ (0.5 L) was added to 0.5 kg of RH (previously milled to 0.5 mm), and the digestion occurred for 2 h at 60 °C. The digested husk was then filtered (mesh 85 µm) and washed with distilled water until pH ca.7. The neutralized husk was then dried at 110 °C for 12 h. The SiO_2_ particles (SiO_2__AD) were recovered after a calcination step (the temperature was raised to 700 °C at 4.5 °C/min and maintained at 700 °C for 3.5 h).
- Alkaline extraction: RH was calcined at 700 °C (4.5 °C/min up to 700 °C and maintained at 700 °C for 3.5 h). Then, 10 g of RH ash was added to 0.2 L of NaOH aqueous solution (2.5 M) and refluxed at 90 °C for 3 h. The mixture was then filtered, and the supernatant was recovered and neutralized with HCl 10%. After 24 h, the obtained gel was centrifuged at 9000 rpm for 10 min and washed with water. The washing and centrifugation steps were repeated 3 times, and the obtained SiO_2_ particles (SiO_2__SG) were dried at 150 °C for 2 h.

The SiO_2_ particles obtained by acidic extraction were modified with QAS and PA. In brief, a dispersion (1.5 wt.%) of SiO_2__RH_AD particles in EtOH:H_2_O (1:1) was prepared. PA was added to the mixture, and after homogenization, QAS was added dropwise (molar ratio SiO_2__RH_AD:PA:QAS was 1:5:1.3). The reaction was kept at 23 °C for 3 h, and the as-obtained solid was centrifuged (10 min at 9000 rpm) and washed 3 times with water. After this process, the agglomerates of particles were dried at 40 °C and ground to obtain the particles as a powder (SiO_2__AD@QAS-PA).

The main features of the synthesized SiO_2_ particles are summarized in Table 4.

#### 4.2.2. Synthesis of SiO_2_-Doped Polyurethane Aerogels

SiO_2_-doped PUR aerogels were prepared at a pilot-plant scale (using a freeze-drying unit with a production of 2 m^2^ of aerogel panels per batch), following our previous reported works [42,43]. The main modification involved the incorporation of a doping agent (SiO_2_ particles). Table 5 lists the prepared aerogel samples.

During the synthesis, the different types of silica particles extracted from RH were doped in the PUR aerogels. PEG (polyol, 5.23·10^−2^–5.54·10^−2^ mol) and DMPA (emulsifier agent, 0.17 mol) that were previously conditioned for dehydration are used. SiO_2_ particles of specific types (SiO_2__AD, SiO_2__SG, SiO_2__AD@QAS-PA) were added at a concentration of 2 wt.% for all types except for SiO_2__AD particles, which ranged from 0.5 to 3.0 wt.%.

PEG and DMPA were melted together with SiO_2_ particles under vigorous mechanical stirring in a jacketed vessel at 80 °C. The amount of PEG was adjusted for each experiment based on the amount of doping agent added. Once melted, HMDI (isocyanate, 0.34 mol), NMP (anhydrous solvent, 0.17 mol), and DBTDL (catalyst, 8.86·10^−4^ mol) were added, and the reaction proceeded for 3 h at 450 rpm, maintaining the same temperature. Subsequently, the temperature was lowered to 40 °C before adding EtOAc (organic solvent, 4.04 mol). The mixture was stirred for an additional hour before adding TEA (neutralizer agent, 0.20 mol) to neutralize the DMPA acidic functionalities. This neutralization step took 30 min to achieve complete neutralization of carboxylic groups. All previous steps were carried out under a N_2_ stream to create an inert atmosphere. In the next step, polymer chains were extended using a slight excess of the chain extender EDA (0.07 mol), adding another 30 min to the process. The EDA/HMDI ratio was 0.20. In the final stage of the synthesis method, water content was added, and the polymer dispersion was completed by mechanical stirring (900 rpm) for approximately 30 min. The added water content affected the solid content of the samples, fixed at 3.7 wt.%. Finally, the obtained wet gel was transferred into the freeze-drying trays with dimensions of 43.5 cm (long) × 34 cm (wide) × 4 cm (high) and subjected to a freeze-drying process involving freezing for 6 h at −40 °C, followed by 60 h at 25 °C and 200 µbar of primary drying and, finally, 10 h at 40 °C of secondary drying.

### 4.3. Characterization Techniques

Thermogravimetric analyses of SiO_2_ particles and SiO_2_-doped polyurethane aerogels (TGA, 2STARe system, Mettler Toledo, Madrid, Spain) were conducted to assess their thermal stability. Additionally, the glass transition temperature of PUR aerogels (*T*_g_) was determined through differential scanning calorimetry (DSC, 2STARe system, Mettler Toledo, Madrid, Spain) at a heating rate of 20 K·min^−1^, spanning from −80 to 80 °C. Data acquisition for both TGA and DSC experiments was performed using 2STARe V16.30 software.

The chemical structure of the samples was determined by assigning the functional groups based on vibrations obtained with an infrared spectrometer (Fourier transform infrared–attenuated total reflectance (FTIR–ATR), Spectrum Two, PerkinElmer, Waltham, MA, US) in the 500–4000 cm^−1^ range.

Density values of the synthesized aerogels were determined using a 3D scanner (REXCAN DS3 Silver, eQuality Tech Inc., Rochester Hills, MI, USA). The instrument generated a three-dimensional image of the sample with ezScan v 3.26 software, and the volume of the aerogel was subsequently calculated using Geometric Wrap v2021.2.2 software. Specifically, three square-shaped samples were extracted from different regions of each synthesized aerogel. The samples were previously weighted to calculate their density (ρ, g·cm^−3^) by dividing their mass by the volume obtained from the scanner.

Moreover, the thermal conductivity of the synthesized aerogels was assessed through heat transfer measurements between two parallel plates (HFM 300, Linseis, Selb, Germany). To conduct these assessments, samples with dimensions of 30 × 30 cm were used. These analyses were carried out at five intermediate temperatures, between 0 and 40 °C.

The morphological structure of the samples was examined through high-resolution scanning electron microscopy (HRSEM, GeminiSEM 500, ZEISS, Jena, Germany) using an 80 mm^2^ energy dispersive spectroscopy (EDS) sensor and another electron backscatter diffraction (EBSD) sensor. Thus, SEM images, elemental mapping, and EDS spectrum were obtained.

To analyze the pore size distribution (PSD) of the aerogels, a PoreMaster mercury-intrusion apparatus (Quantachrome Instruments, Boynton Beach, FL, USA) was employed. The method relies on the infiltration of mercury into the pores of the samples under controlled pressure, enabling penetration. Subsequently, the registration of mercury volume, correlated with increasing pressure, allows the determination of the pore size distribution [44].

Finally, the flame-retardancy behavior of the synthesized aerogels was directly assessed in the absence and presence of the different SiO_2_ particles (AD, SG, and AD@QAS-PA). The flame spread across vertically oriented aerogels (ca. 10 × 3 cm) was evaluated according to [45]. The ignition source was applied to the aerogel surface for 3 s, followed by a waiting period of 10 s to measure the subsequent flame spread. Each analysis was carried out with triplicate samples (*n* = 3).

## Figures and Tables

**Table 1:** Characterization results of the SiO_2_-doped PUR aerogels.

Sample	SiO_2_ Particles Density (g·cm^−3^)	Surface Area (m^2^·g^−1^) *	Total Intruded Pore Volume (cc·g^−1^) *	Density (±0.002, g·cm^−3^)	Thermal Conductivity (±0.0010, W·m^−1^·K^−1^)	Young’s Modulus (±0.0005, MPa)	*T*_g_ (°C)
PUR	-	11.67	3.17	0.051	0.0387	0.001	−49.8
PUR_AD_2_	0.30	13.48	4.71	0.058	0.0346	0.005	−48.9
PUR_SG_2_	0.32	0.36	2.92	0.068	0.0346	0.013	−47.2

* Obtained by a Hg porosimeter.

**Table 2:** Characterization results of the SiO_2_-doped PUR aerogels with different particle contents.

Sample	Surface Area (m^2^·g^−1^) *	Total Intruded Pore Volume (cc·g^−1^) *	Density (±0.002, g·cm^−3^)	Thermal Conductivity (±0.0010, W·m^−1^·K^−1^)	Young’s Modulus (±0.0005, MPa)
PUR_AD_0.5_	0.42	2.92	0.064	0.0363	0.001
PUR_AD_1_	9.68	3.14	0.063	0.0338	0.002
PUR_AD_2_	13.48	4.71	0.058	0.0346	0.005
PUR_AD_3_	11.14	4.07	0.056	0.0357	0.008

* Obtained by a Hg porosimeter.

**Table 3:** Characterization results of the SiO_2_-doped PUR aerogels with particles derived from acidic extraction.

Sample	SiO_2_ Particle Density (g·cm^−3^)	Surface Area (m^2^·g^−1^) *	Total Intruded Pore Volume (cc·g^−1^) *	Density (±0.002, g·cm^−3^)	Thermal Conductivity (W·m^−1^·K^−1^)	Young’s Modulus (±0.0005, MPa)	*T*_g_ (°C)
PUR_AD_2_	0.30	13.48	4.71	0.058	0.0346	0.005	−48.9
PUR_AD@QAS-PA_2_	0.53	0.36	2.06	0.065	0.0335	0.010	−47.5

* Obtained by a Hg porosimeter.

**Table 4:** Main characteristics of the extracted SiO_2_ particles from rice husk.

	SiO_2_ Particle Type		
	AD	AD@QAS-PA	SG
Method of production	Acidic extraction	Acidic extraction Functionalization with PA and QAS	Alkaline extraction Sol–gel precipitation
Inorganic residue (%)—evaluated by TGA analysis (800 °C)	98.7	39.6	88.6
Z potential (mV)	−20 ± 1 (water)	41 ± 2 (ethanol)	−34 ± 1 (water)
Morphology (SEM images)	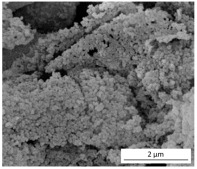	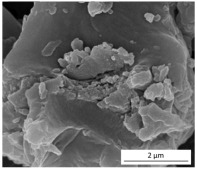	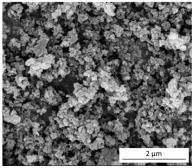
Elemental composition	C (9 wt.%) O (44 wt.%) Si (47 wt.%)	C (54 wt.%) O (23 wt.%) Si (10 wt.%) P (8 wt.%) impurities (5 wt.%)	C (3 wt.%) O (59 wt.%) Si (32 wt.%) impurities (6 wt.%)
Density (g·cm^−3^)	0.30	0.53	0.32

**Table 5:** SiO_2_ nature and content of the final doped PUR aerogels.

Set	Experiment ^(^*^)^	PUR Sample ^(^**^)^	Doping Agent	SiO_2_ Content (wt.%)
	0	PUR	None	-
1	1	SiO_2__AD_2_	AD	2
	2	SiO_2__AD@QAS-PA_2_	AD@QAS-PA	
	3	SiO_2__SG_2_	SG	
2	4	SiO_2__AD_0.5_	AD	0.5
	5	SiO_2__AD_1_		1
	6	SiO_2__AD_2_		2
	7	SiO_2__AD_3_		3

^(^*^)^ Experiments labeled 1 and 6 are identical. ^(^**^)^ SiO_2__AD*_x_*: silica obtained by acidic-extraction-based PUR aerogel containing *x* wt.% of silica related to the total amount of PUR. SiO_2__AD@QAS-PA*_x_*: silica obtained by acidic extraction and functionalized with dimethyloctadecyl(3-(trimethylsilyl)propyl ammonium chloride and phytic-acid-based PUR aerogel containing *x* wt.% of silica related to the total amount of PUR. SiO_2__SG*_x_*: silica obtained by alkaline extraction, followed by sol–gel-precipitation- based PUR aerogel containing *x* wt.% of silica related to the total amount of PUR.

## Data Availability

The data presented in this study are openly available in article.

## Supplementary Materials

The following supporting information can be downloaded at https://www.mdpi.com/article/10.3390/gels10070465/s1. Figure S1: EDS analysis and elemental mapping of PUR_AD2 sample; Figure S2: FTIR spectra corresponding to the different silica particles; Figure S3: TGA curves relating to the three types of SiO_2_ particles; Figure S4: Combustion process of reference aerogel (undoped) and aerogel doped with 2% AD_SiO_2_ particles. Inset: shows the images obtained using a thermal camera; Figure S5: (a) Main reactions and (b) mechanism for the thermal degradation of polyurethane (PUR). References [46,47,48,49,50,51,52,53,54,55] are cited in the Supplementary Materials.
