# Supplementary material for: Enhancing Flame-Retardant Properties of Polyurethane Aerogels Doped with Silica-Based Particles
Source: Gels. 2024 Jul 16;10(7):465. doi: 10.3390/gels10070465 (PMC11276070; doi:10.3390/gels10070465)
Supplement: Supplementary file 1 [file gels-10-00465-s001.zip › gels-3092241-supplementary.pdf]

Supplementary Material

Enhancing flame-retardant properties of polyurethane aerogels doped with silica-based particles

Esther Pinilla-Peñalver[1], *Óscar del Fresno*[1], *Darío Cantero*[1], *Adriana Moreira*[2], *Filipa Gomes*[2], *Francisca Miranda*[2], *Marcelo Oliveira*[2], *Mariana Ornelas*[2], *Luz Sánchez-Silva*[1] *and Amaya Romero*[1]*

[1]Department of Chemical Engineering, University of Castilla-La Mancha

Avda. Camilo José Cela 12, 13071 Ciudad Real, Spain

[2]CeNTI – Centre for Nanotechnology and Smart Materials, Rua Fernando Mesquita 2785, 4760-034 V. N. Famalicão, Portugal

*Corresponding author e-mail: amaya.romero@uclm.es

RESULTS AND DISUSSION

Characterization of silica particles

In this work, three different types of SiO_2-based particles were evaluated as doping agents for aerogels, namely SiO_2_AD, SiO_2_SG and SiO_2_AD@QAS-PA. The main features of the synthesised silica particles are summarized in **Table 4** although they will be described in detail below.

Scanning electron microscopy (SEM) micrographs for the three different samples are also shown in **Table 4** revealing the morphological characteristics of the particles, as well as the corresponding chemical composition determined by energy-dispersive spectroscopy (EDS). The non-modified SiO_2 particles (SiO_2_AD and SiO_2_SG) exhibited a spherical morphology in the range of 40 – 80 nm. However, with the incorporation of QAS-PA, its morphology changed to a more agglomerated status, probably formed by the self-condensation of QAS, as suggested in the work by Gong et al. [1]. Nevertheless, the diameter of the individual particles did not change significantly. The EDS analysis confirmed the increase of C content and the appearance of P in SiO_2_AD@QAS-PA particles. These elements are characteristic from dimethyloctadecyl(3-(trimethylsilyl)propyl ammonium chloride solution (QAS) and phytic acid (PA), respectively, indicating that the modification of the particles was successful.

Figure S2 shows the Fourier-transform infrared (FTIR) spectra of the different SiO_2 particles. As expected, the spectra for all particles exhibited bands at ca. 800 and 1050 cm^{-1}, which can be assigned to the symmetric and asymmetric stretching vibration of Si-O-Si bonds, respectively [2, 3]. Comparing the FTIR spectrum of SiO_2_AD with the spectrum of SiO_2_SG, it is possible to see the presence of a silanol group (stretching vibration around 954 cm^{-1}) when the silicas are obtained by alkaline method, due the sol-gel process like Stöber synthesis of SiO_2 [4].

For the SiO_2_AD@QAS-PA particles, a broad band at 2110 – 2480 cm^{-1} is can also be observed, corresponding to the OH—P=O groups from PA [5]. The presence of characteristic peaks of C-H stretching and deformation vibration bands at 2917, 2852, and 1467 cm^{-1} from QAS [6] also confirms the modification of the silica particles.

The FTIR spectrum in **Figure S2** exhibited characteristic peaks of SiO_2 framework, particularly at 1103 cm^{-1} for the O-Si-O asymmetric stretching vibration and, at 794 cm^{-1} for the O-Si-O symmetric stretching vibration [7]. FTIR spectrum of SiO_2_AD did not show any more peaks confirming the absence of other organic and inorganic materials that demonstrated high purity of the obtained SiO_2 particles. The SiO_2_SG shows a characteristic peak of silanol group (Si-OH) stretching vibration at around 954 cm^{-1} due to the sol-gel process similar to Stöber SiO_2 particles [4].

The peak at 1629 cm^{-1} displayed by SiO_2_AD@QAS-PA and SiO_2_SG particles was assigned to H-O-H bending vibrations of trapped water molecules in the SiO_2 matrix [8]. The presence of characteristic peaks of C-H stretching and deformation vibration bands at 2917, 2852, and 1467 cm^{-1} from QAS also confirms the modification of the SiO_2 particles.

In **Table 4** the values obtained for the charge of the particles evaluated by zeta potential are reported. Non-modified silica particles exhibited a negative zeta potential; the values obtained for SiO_2_SG are similar to the zeta potential of Stöber particles (-34 mV). Due to their inherent hydrophobicity, the SiO_2_AD@QAS-PA particles had to be analyzed in an ethanol dispersion. As expected, the modification of SiO_2 with PA-QAS increased the zeta potential to a more positive value, in line with what was previously reported by Wang et al. [9].

The results obtained for the thermogravimetric analysis (TGA) of the different SiO_2 particles are shown in **Figure S3**. The residual mass observed for both SiO_2_AD and SiO_2_SG (98.7% and 88.6%, respectively) confirms the inorganic nature of the non-modified SiO_2 particles extracted from rice husk (RH). The observed differences in the remaining percentage are justified by the different methods used to obtain the particles; indeed, the SiO_2_AD particles are obtained after a calcination step, that eliminates the hydroxyl groups from the surface of the SiO_2 particles. On the other hand, the SiO_2_SG particles do not undergo this calcination step. As a result, silanol groups are present on the surface, leading to the observed water loss for these particles at approximately 100 °C. In short, it can be concluded that both the RH acid and alkaline extraction produces SiO_2 particles (acid digestion (AD) and sol gel (SG)) of high purity and therefore very thermally resistant.

Modification of the AD particles with QAS (AD@QAS-PA) results on a much less Si content and thermal stability up to 230 ºC structures. On what regard the particles SiO_2_AD@QAS-PA, the observed residual mass is 39.6%. This low value confirms the modification of the silica particles with the organic groups of QAS, as the loss in the temperature range of around 200 - 300 °C is characteristic for the quaternary amine compounds, as reported by Galimberti et al. [10]. (PA) also decomposes at different temperatures due to processes of dehydration and carbonization, for temperatures below 550 °C, and to char degradation (above 550 °C).

Figures and Tables

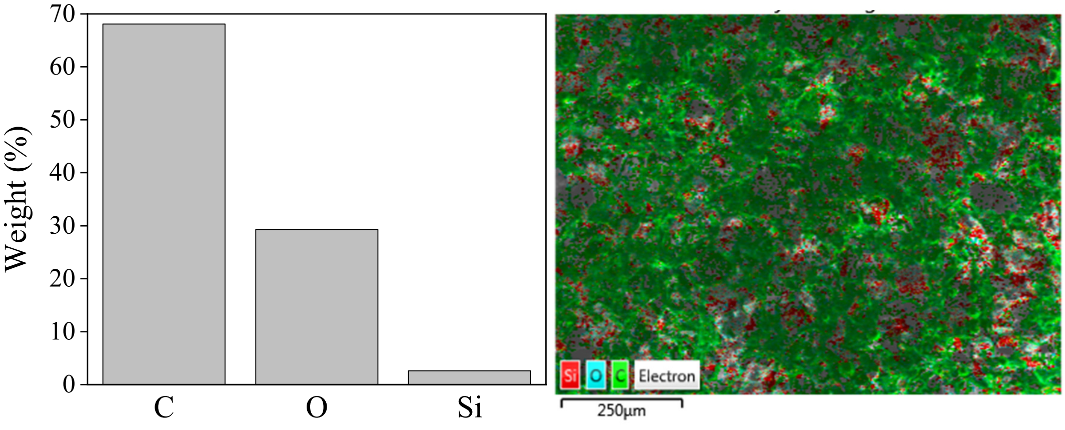

Figure S1. EDS analysis and elemental mapping of PUR_AD_2 sample.

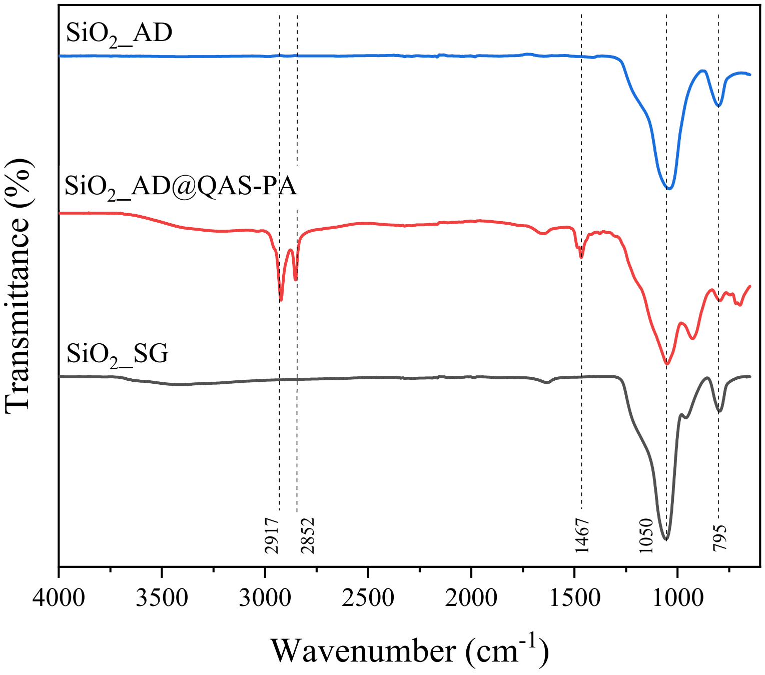

Figure S2. FTIR spectra corresponding to the different silica particles.

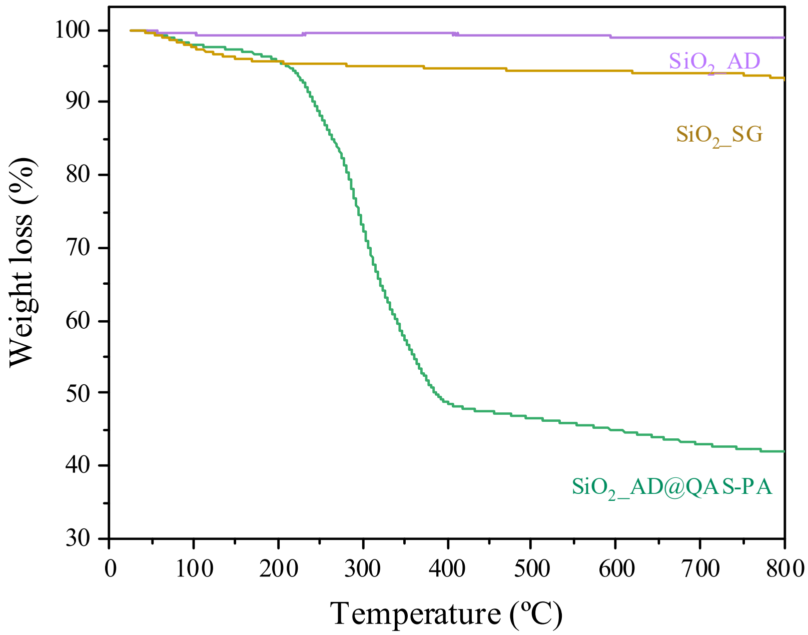

Figure S3. TGA curves relating to the three types of SiO_2 particles.

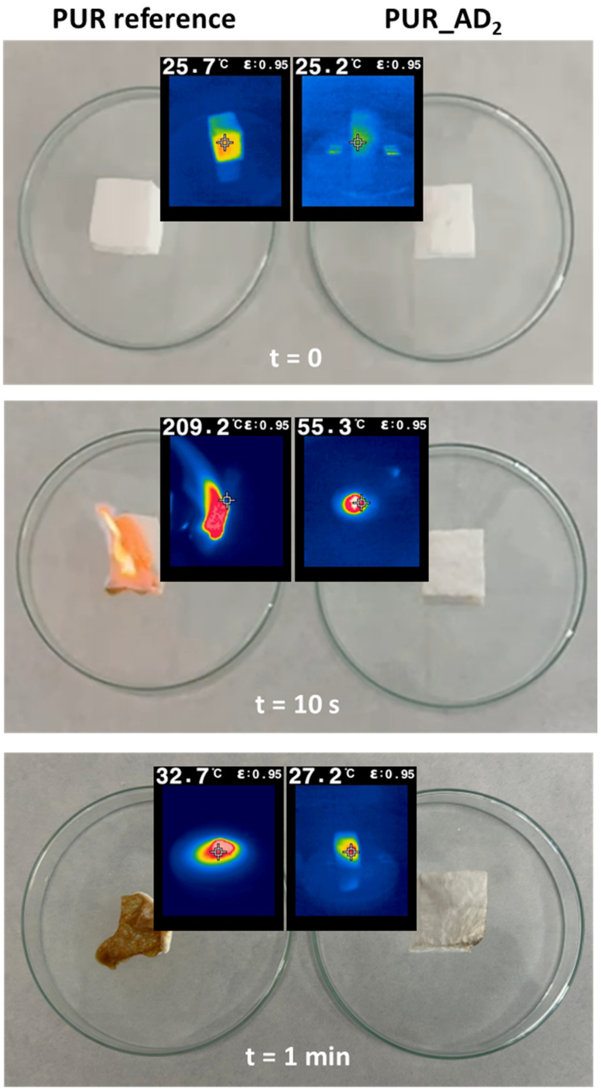

Figure S4. Combustion process of reference aerogel (undoped) and aerogel doped with 2% AD_SiO_2 particles. Inset: shows the images obtained using a thermal camera.

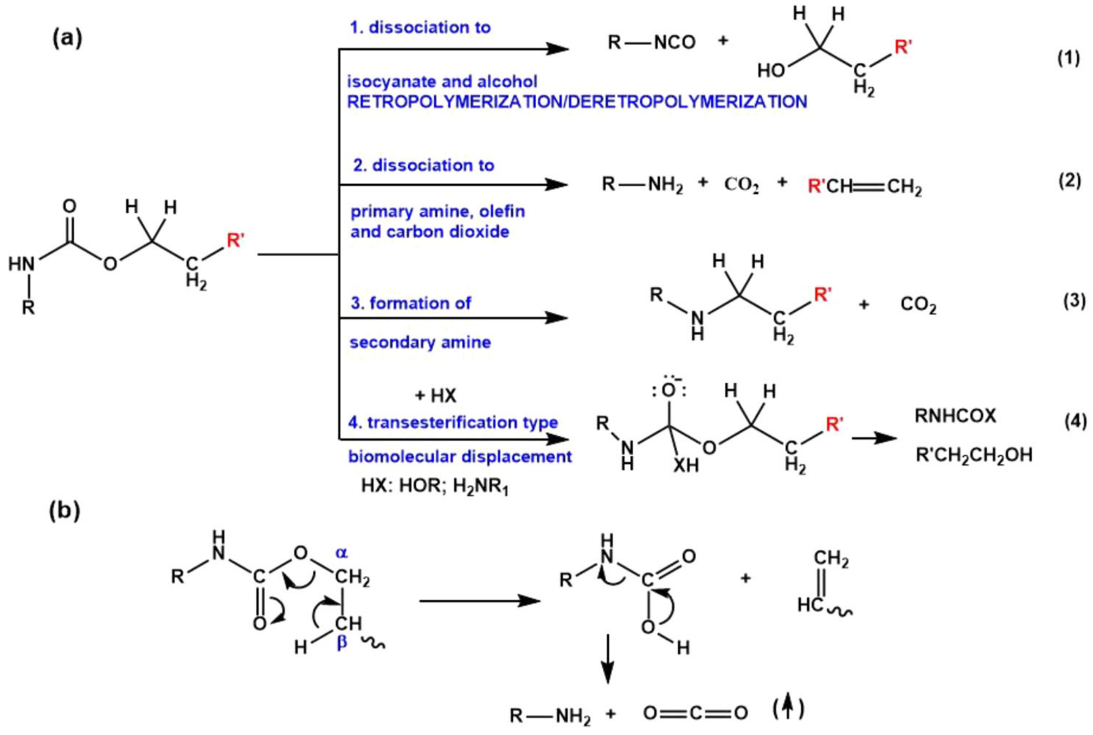

Figure S5. (a) Main reactions and (b) mechanism for the thermal degradation of polyurethane (PUR) [11].

References

[1] Gong, S.Q.; Epasinghe, D.J.; Zhang, W.; Zhou, B.; Niu, L.N.; Ryou, H.; Eid, A.A.; Frassetto, A.; Yiu, C.K.Y.; Arola, D.D.; Mao, J.; Pashley, D.H.; Tay, F.R. Synthesis of antimicrobial silsesquioxane–silica hybrids by hydrolytic co-condensation of alkoxysilanes. *Polym. Chem.* **2014**, *5*(2), 454-462. https://doi.org/10.1039/C3PY00635B.

[2] Li, K.M.; Jiang, J.G.; Tian, S.C.; Chen, X.J.; Yan, F. Influence of silica types on synthesis and performance of amine–silica hybrid materials used for CO_2 capture. J. Phys. Chem. C **2014**, *118*(5), 2454-2462. https://doi.org/ 10.1021/jp408354r.

[3] Ullah, R.; Deb, BK.; Mollah, M.Y.A. Synthesis and characterization of silica coated iron-oxide composites of different ratios. *Int. J. Compos. Mater.* **2014**, *4*(2), 135-145. https://doi.org/10.5923/j.cmaterials.20140402.13.

[4] Comite, A. Preparation of silica membranes by sol-gel method. In: *Current trends and future developments on (bio-)membranes,* 1st ed.; Basile, A; Favvas, E.P.; Elsevier, Amsterdam, Netherlands, 2017; pp. 3-23.

[5] Zhang, R.; Cai, S.; Xu, G.; Zhao, H.; Li, Y.; Wang, X.; Huang, K.; Wu, X. Crack self-healing of phytic acid conversion coating on AZ31 magnesium alloy by heat treatment and the corrosion resistance. *Appl. Surf. Sci.* **2014**, *313*, 896-904. https://doi.org/10.1016/j.apsusc.2014.06.104.

[6] Yudovin-Farber, I.; Beyth, N.; Weiss, E.I.; Domb, A.J. Antibacterial effect of composite resins containing quaternary ammonium polyethyleneimine nanoparticles. *J. Nanopart. Res.* **2010**, *12*, 591-603. https://doi.org/10.1007/s11051-009-9628-8.

[7] Dang, N.T.T.; Nguyen, T.T.A.; Phan, T.D.; Tran, H.; Van Dang, P.; Nguyen, H.Q. Synthesis of silica nanoparticles from rice husk ash. *Sci. Technol. Develop. J.* **2017**, *20*(K7), 50-54. https://doi.org/10.32508/stdj.v20iK7.1211.

[8] Li, Y.; Lan, J.Y.; Liu, J.; Yu, J.; Luo, Z.; Wang, W.; Sun, L. Synthesis of gold nanoparticles on rice husk silica for catalysis applications. *Ind. Eng. Chem. Res.* **2015**, *54*(21), 5656-5663. https://doi.org/10.1021/acs.iecr.5b00216.

[9] Wang, X.; Shi, L.; Zhang, J.; Cheng, J.; Wang, X. Self-assembly fabrication, microstructures and antibacterial performance of layer-structured montmorillonite

nanocomposites with cationic silica nanoparticles. *RSC Adv.* **2017**, *7*(50), 31502-31511. https://doi.org/10.1039/C7RA04353H.

[10] Galimberti, M.; Martino, M., Guenzi, M.; Leonardi, G.; Citterio, A. Thermal stability of ammonium salts as compatibilizers in polymer/layered silicate nanocomposites, e-*Polymers* **2009**, *9*(1), 56-69. https://doi.org/10.1515/epoly.2009.9.1.686.

[11] Chattopadhyay, D.K.; Webster, D.C. Thermal stability and flame retardancy of polyurethanes. *Prog. Polym. Sci.* **2009**, *34*(10), 1068-1133. https://doi.org/10.1016/j.progpolymsci.2009.06.002.
